# Measuring Heat Stress for Human Health in Cities: A Low-Cost Prototype Tested in a District of Valencia, Spain

**DOI:** 10.3390/s23229285

**Published:** 2023-11-20

**Authors:** Àlex Aduna-Sánchez, Antonio Correcher, David Alfonso-Solar, Carlos Vargas-Salgado

**Affiliations:** 1 Institute for Energy Engineering, Universitat Politècnica de València, Camí de Vera s/n, 46022 Valencia, Spain; alsanad@doctor.upv.es (À.A.-S.); daalso@iie.upv.es (D.A.-S.); carvarsa@upvnet.upv.es (C.V.-S.); 2 Instituto de Automática e Informática Industrial, Universitat Politècnica de València, Camino de Vera s/n, 46022 Valencia, Spain

**Keywords:** heat stress, WBGT, MRT, TSI, PHS, Arduino, smart city, low-cost

## Abstract

Nowadays, the measurement of heat stress indices is of principal importance due to the escalating impact of global warming. As temperatures continue to rise, the well-being and health of individuals are increasingly at risk, which can lead to a detrimental effect on human performance and behavior. Hence, monitoring and assessing heat stress indices have become necessary for ensuring the safety and comfort of individuals. Thermal comfort indices, such as wet-bulb globe temperature (WBGT), Tropical Summer Index (TSI), and Predicted Heat Strain (PHS), as well as parameters like mean radiant temperature (MRT), are typically used for assessing and controlling heat stress conditions in working and urban environments. Therefore, measurement and monitoring of these parameters should be obtained for any environment in which people are constantly exposed. Modern cities collect and publish this relevant information following the Smart City concept. To monitor large cities, cost-effective solutions must be developed. This work presents the results of a Heat Stress Monitoring (HSM) system prototype network tested in the Benicalap-Ciutat Fallera district in Valencia, Spain. The scope of this work is to design, commission, and test a low-cost prototype that is able to measure heat stress indices. The Heat Stress Monitoring system comprises a central unit or receiver and several transmitters communicating via radiofrequency. The transmitter accurately measures wind speed, air temperature, relative humidity, atmospheric pressure, solar irradiation, and black globe temperature. The receiver has a 4G modem that sends the data to an SQL database in the cloud. The devices were tested over one year, showing that radio data transmission is reliable up to 700 m from the receiver. The system’s power supply, composed of a Photovoltaic panel and Lithium-ion batteries, provided off-grid capabilities to the transmitter, with a tested backup autonomy of up to 36 days per charge. Then, indicators such as WBGT, TSI, and MRT were successfully estimated using the data collected by the devices. The material cost of a 12-point network is around EUR 2430 with a competitive price of EUR 190 per device.

## 1. Introduction

Institutions and researchers around the world report rises in the Earth’s surface temperature year by year [1,2,3]. Global warming concerns governments that signed the Paris Agreement in 2015 to keep the temperature rise under 2 °C. The effects of global warming are well known as extreme weather events, polar ice melting, and increases in average temperatures. Increasing the average temperature in populated areas can cause heat stress over the population at specific zones and times. That is relevant because it directly affects people’s health [4,5] and has a significant economic impact [6].

Heat Stress (HS) is a known phenomenon that can be parametrized, and its monitoring can help to improve the citizens’ quality of life. HS occurs when a body cannot expel excess heat. When that happens, people feel dizzy, have a high heart rate, have muscle cramps, and have an increased risk of heatstroke. HS is usually monitored to guarantee safe indoor and outdoor working conditions [7]. Still, it is also essential to study healthy living conditions in populated areas [8]. This is particularly critical in cities, as urban microclimate conditions can cause higher heat stress outdoors, as well as having a direct influence on thermal conditions inside the buildings [9,10].

Modern cities are evolving to the Smart City concept [11,12], making relevant information available to citizens and governors. In that direction, many researchers are developing sensor networks to gather details about city performance [12,13,14,15]. As stated by Bacco et al. [16], environmental monitoring is a key issue in Smart Cities as it can effectively monitor air quality [17] and thermal comfort.

However, many wireless sensors are needed to monitor a city thoroughly, so cost-effective solutions must be developed [18]. In this sense, recent research explores the cost reduction in Smart City monitoring applications. For example, Almalki et al. [19] presented a sensor architecture for Smart Cities to reduce the energy consumption of each node in the network. Another approach to lowering the cost of measuring data is using non-dedicated sensing systems (such as mobile phones) to increase the number of nodes [20] and merge this information with traditional sensing nodes. Nevertheless, some measures, such as humidity, wind speed, and wind direction (among others), are complicated to measure from non-dedicated nodes. A low-cost sensor for meteorological purposes is presented in Nan et al. [21]. The sensor can be deployed as a node in the measurement network. The variables it measures are temperature, humidity, and pressure, but the authors report low operating time and weaknesses against extreme weather conditions. Another approach oriented to low-cost sensors is presented by Hashmy et al. [22]. In this case, the authors do not show a device but rather a calibration method for low-cost sensors that increase the reliability of the measures obtained from low-cost devices.

ISO 7726 [23], ISO 7933 [24], and ISO 7243 [25] regulations standardize the methods for defining and measuring a heat stress index. The most used parameter to measure heat stress is the wet-bulb globe temperature (WBGT) [26,27]. Other commonly used indices are the Predicted Heat Strain (PHS) [28], the Universal Thermal Climate Index (UTCI) [29], the Tropical Summer Index (TSI), and the Thermal Working Limit (TWL). Other parameters such as the mean radiant temperature (MRT) [30] can also be helpful. In any case, to state the current HS, environmental variables (temperature, humidity, etc.) must be continuously measured. When studying HS in cities, a net of reliable measuring nodes will be needed.

Generally, commercial devices compute WBGT to characterize HS, although most modern HS meters give other indicators such as TWL. To calculate WBGT, they measure several magnitudes: air temperature, relative humidity, black globe temperature, radiant sunlight, or air pressure. They usually use chargers or batteries as the power source. In the standalone power case, the device autonomy can reach 16 days at the best chance. Regarding data logging, not all devices allow this feature. Those allowing data storage use custom applications to show data and do not allow real-time connection with external systems. Customers can find HS meters in the market, ranging from EUR 250 to EUR 800. Moreover, high-precision HS meters are available at prices reaching EUR 5000, turning these options too expensive if a large network with multiple nodes is needed or not suitable for outdoor urban environments. Some low-cost dedicated solutions to measure heat stress have already been developed, such as the one presented in Sulzer et al. [31]. In that case, a very cost-effective solution was obtained, but it required on-grid power supply and W-Fi connection, which are not always available.

Heat stress numerical modeling represents a different but complementary approach to measurement systems for evaluating and managing heat stress. Measurement systems directly assess physiological responses, such as core body temperature and heart rate, providing real-time and accurate data on individuals’ conditions. This approach allows for immediate interventions, catering to individual variability and offering concrete validation of heat stress in specific environments. On the contrary, heat stress modeling relies on computer simulations to predict heat stress levels based on environmental factors, enabling proactive planning and cost-effective assessments across diverse scenarios. While measurement systems excel in precision and real-time action, modeling offers predictive capabilities and cost efficiency, making the choice between them dependent on the specific goals and context of the application.

Currently, several numerical models are employed to simulate the heat stress index. PALM [32] (Parallelized Large-Eddy Simulation Model) was crafted for simulating atmospheric and environmental flows. It has gained widespread usage in investigating heat stress in urban areas [33], taking into account factors such as radiation [34,35], convection, and the thermal properties of surfaces. Its capability to simulate complex urban structures adds depth to its applications. Some authors have developed works using PALM [36,37]. In contrast, ENVI-met [38] (Environmental Meteorology) is a microscale meteorological model primarily focusing on simulating urban microclimates. This model considers intricate interactions between buildings, vegetation, and the atmosphere, enabling the modeling of temperature, humidity, and other meteorological parameters at high spatial and temporal resolutions. ENVI-met proves valuable in assessing the impact of urban planning and design on heat stress, offering insights that help optimize green spaces and ventilation to create more comfortable urban environments. Works carried out using ENVI-met have been developed by [39]. Another tool is MITRAS [40] (Microscale Transport and Stream model), a comprehensive urban climate model developed to analyze the effects of different urban structures and materials on local climate conditions. MITRAS considers factors such as radiation, heat conduction, and airflow to simulate the thermal behavior of urban surfaces. It plays a pivotal role in evaluating the thermal performance of building materials and urban layouts, aiding in the design of energy-efficient and heat-resilient urban areas. Salim et al. [41] describes the developing theory and underlying processes of the microscale obstacle-resolving model MITRAS. Finally, RayMan [42], developed by the Meteorological Institute at the University of Freiburg, Germany, is designed for assessing solar radiation and thermal comfort. RayMan calculates human-biometeorological parameters, including the physiological equivalent temperature (PET), to measure thermal comfort. The RayMan model considers variables such as solar radiation, wind speed, and humidity to estimate the thermal sensation and potential heat stress experienced by individuals in outdoor environments. Human Thermal Comfort and heat stress models in urban areas using RayMan are analyzed in [43,44]. While these models significantly contribute to developing effective strategies for mitigating heat stress, it is essential to underscore the necessity for real measurements. Integrating these models with on-the-ground observations is crucial for comprehensively understanding and implementing strategies to combat heat stress in diverse environments and comprehensive means to assess such parameters.

This paper shows a cost-effective proposal for heat stress index measuring in outdoor environments. The Heat Stress Monitoring (HSM) system prototype can be deployed as a sensor network, thus covering broad areas, and it is battery-powered, featuring interesting off-grid and wireless capabilities.

## 2. Methodology

### 2.1. Heat Stress Indices

This section describes some of the heat stress indicators that can be directly calculated from environmental signals measured by the prototype proposed in this paper. It also shows other examples of the most used heat stress indices that require these parameters although they are not directly computable.

#### 2.1.1. Wet-Bulb Globe Temperature (WBGT)

According to ISO 7243 [25], to obtain the WBGT index (in °C), there are two possible situations: WBGT index calculation for indoor (or outdoor without solar irradiance) and outdoor (with solar irradiance) environments. These two cases are calculated by Equation (1) and Equation (2), respectively.

(1) $${WBGT}_{indoor}=0.7t_{nwb}+0.3t_{g}$$

(2) $${WBGT}_{outdoor}=0.7t_{nwb}+0.2t_{g}+0.1t_{a}$$

where $t_{nwb}$ (°C) is the natural wet-bulb temperature, $t_{g}$ (°C) is the globe temperature, and $t_{a}$ (°C) is the dry air temperature. $t_{nwb}$ is measured by a thermometer with its bulb covered with a wettened cotton, without shields for avoiding wind or radiation and under natural ventilation conditions. It can be calculated using Equation (3) [45].

(3) $$t_{nwb}=t_{w}+0.0021S-0.42v_{w}+1.93$$

where, $t_{w}$ (°C) is the psychometric temperature of wet bulb, S (W·m^−2^) is solar irradiation, and $v_{w}$ (m·s^−1^) is the wind speed. $t_{w}$ can be calculated with Equation (4), using air temperature ($t_{a}$) and relative humidity (RH%) measurements at standard sea level, as proposed by Stull [46].

(4) tw=tatan−1⁡0.151977RH%+8.13165912+tan−1⁡ta+RH%−tan−1⁡RH%−1.676331+0.00391838RH%32·tan−1⁡0.023101RH%−4.686035

#### 2.1.2. Tropical Summer Index (TSI)

TSI is defined as the temperature of calm air at 50% relative humidity which imparts the same thermal sensation as the given environment [47]. TSI (in °C) is calculated using Equation (5), where $t_{g}$ is the globe temperature (°C).

(5) $$TSI=0.745t_{g}+0.308t_{nwb}-2.06\sqrt{v_{w}+0.841}$$

#### 2.1.3. Other Heat Stress Indices

Other heat stress indicators require environmental parameters measured by the device proposed. For example, both the Predicted Heat Strain (PHS) model [24] and the Physiological Equivalent Temperature (PET) [48] require air temperature, mean radiant temperature (MRT), partial vapor pressure, and wind speed. Thermal Working Limit (TWL) uses dry air, wet bulb and globe temperatures, wind speed, and atmospheric pressure [49]. The Universal Thermal Climate Index (UTCI) can be derived with a model that uses air temperature, wind speed at 10 m, relative humidity, and MRT [50].

### 2.2. Mean Radiant Temperature (MRT)

MRT is a key parameter in the calculation of several of the heat stress indices described in this paper. MRT ${\bar{t}}_{r}$ (in °C) is calculated according to ISO 7726 [23] using Equations (6)–(9) depending on the globe diameter and the airflow. Equations (6) and (7) are used for forced and natural convection, respectively, when the globe diameter is less than 15 cm. Equations (8) and (9) are used when the globe diameter is equal to 15 cm. ${\bar{t}}_{r}$ depends on the globe temperature $t_{g}$ (°C), the thermal emissivity of the globe $\varepsilon_{g}$, the diameter of the globe D (m), and the air temperature $t_{a}$ (°C).

(6) t¯rforced=tg+2734+1.1·108vw0.6εg·D0.4tg−ta14−273

(7) t¯r(natural)=tg+2734+0.25·108εgtg−taD14tg−ta14−273

(8) t¯r(forced)=tg+2734+2.5·108vw0.6·tg−ta14−273

(9) t¯r(natural)=tg+2734+0.4·108tg−ta14·tg−ta14−273

The choice between natural or forced convection equations depends on the thermal transfer coefficient value $h_{cg}$ (W·m^−2^·K^−1^). $h_{cg}$ is calculated for forced convection with Equation (10) and for natural convection with Equation (11). If the thermal transfer coefficient for forced convection is greater than that of natural convection, then the forced convection equation for MRT should be used, and vice versa.

(10) $$h_{cg\;(forced)}=6.3\left(\frac{{v_{w}}^{0.6}}{D^{0.4}}\right)$$

(11) hcg (natural)=1.4tg−taD14

### 2.3. Prototype Requirements

This prototype must accurately measure the environmental parameters required to calculate the described heat stress indices: air temperature, relative humidity, wind speed, atmospheric pressure, solar irradiation, and black globe temperature. It may also measure other parameters, such as precipitation and wind direction.

It must also meet the following requirements: operate in indoor and outdoor environments, be powered off-grid, and transmit the collected data wirelessly to a database in the cloud, as well as allowing for local datalogging capability. The whole system will also be designed to be replicable and low-cost.

To test the prototype, several devices will be installed in various locations within the Benicalap-Ciutat Fallera district in Valencia (Spain). The devices will be left to operate for a year, and then the results will be collected.

#### 2.3.1. Wireless Data Transmission Test

The wireless data transmission will be carried out by a 433 MHz radio communication between the measuring device and a receiver. Several transmitters will communicate simultaneously to the same receiver every 15 min. The receiver will then upload the collected data to the cloud using a 4G connection. To test the range and efficiency of the radio system, 30 days of data will be collected. The elapsed time between transmissions will be measured to determine how many failed. Due to the slow dynamics of the studied parameters, the wireless data transmission system will be valid if at least 95% of the measures are taken at most every 35 min. The efficiency of the system will then be calculated using Equation (12).

(12) $$\eta (\%)=\frac{N_{measures\;taken}}{N_{measures\;expected}}\cdot 100$$

Finally, these parameters will be compared with the distances between every transmitter-receiver pair. Thus, the maximum range of the communication system will be obtained for urban environments. Additionally, for this test, several devices will also be installed in Universitat Politècnica de València to test the radio range in line of sight.

#### 2.3.2. Power Supply and Autonomy Test

The energy supply system will be composed of a photovoltaic panel (PV) that will charge up to four 18650 Lithium-Ion batteries, with a capacity of 2.6 Ah each and a rated voltage of 3.7 V, so the capacity of one battery is around 962 Wh if a Depth of Discharge (DoD) of 100% is considered. To obtain the device’s autonomy, it is required to estimate the battery charge level. The state of charge (SoC) is obtained using Equation (13), where V_BAT_ (V) is battery voltage.

(13) $$\left\{\begin{array}{cc} \begin{array}{c} \forall \;V_{BAT}\in \left[4\;\;\;\;\infty \right)\to \\ \forall \;V_{BAT}\in \left[3.1\;\;\;\;4\right)\to \\ \begin{array}{c} \forall \;V_{BAT}\in \left[2.7\;\;\;\;3.1\right)\to \\ \forall \;V_{BAT}\in \left(-\infty \;\;\;2.7\right)\to \end{array} \end{array} & \begin{array}{c} SoC=100\\ SoC=100V_{BAT}-300\\ \begin{array}{c} SoC=25V_{BAT}-67.5\\ SoC=0 \end{array} \end{array} \end{array}\right\}$$

Once the battery capacity is obtained, the device’s energy consumption will be calculated. The autonomy will be estimated with this information and the time for which the device is on. This information was used to size the preliminary battery system. Once the device is installed, an analysis of the autonomy in actual operating conditions will be performed through a test where a device will be left to operate until its battery runs out.

#### 2.3.3. Device Robustness Test

The system will be considered robust if both transmitter and receiver can operate autonomously and without interruptions in indoor and outdoor environments for 12 months. It must endure external conditions such as heavy rain, solar radiation, and extreme weather and be resilient to material degradation. This case will not consider other issues, such as energy shortage. In future developments, if the device is taken to the production phase, it will be sent to certifying entities to certify its water tightness.

## 3. Prototype Design

**Figure 1:**
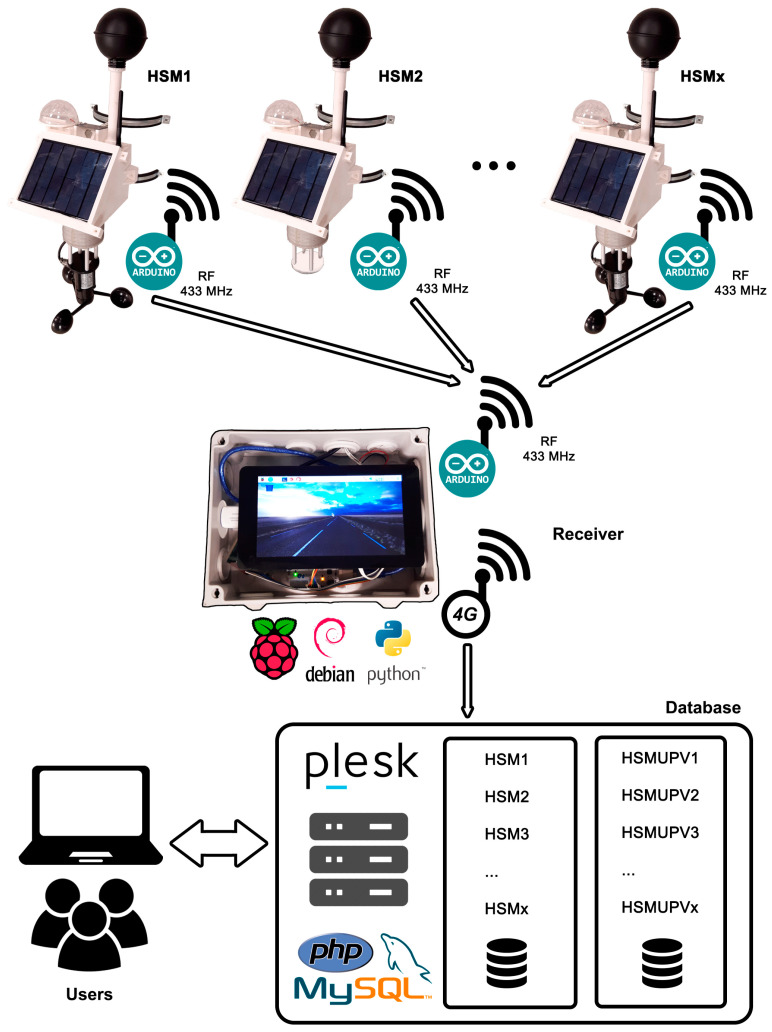
Complete HSM system diagram, including transmitters, receiver, and database.

The Heat Stress Monitoring system (HSM) is a set of devices capable of periodically measuring the environmental parameters necessary to calculate the previous heat stress indices. It consists of two parts. First, a series of devices named transmitters installed in the target locations take measures periodically from their sensors. Then, they send the data through radio transmission to a receiver located in a nearby location. Finally, this receiver collects all the data and uploads it to a database in the cloud through a 4G connection. A diagram of the HSM system is shown in Figure 1.

### 3.1. Transmitter

#### 3.1.1. Transmitter Hardware

**Figure 2:**
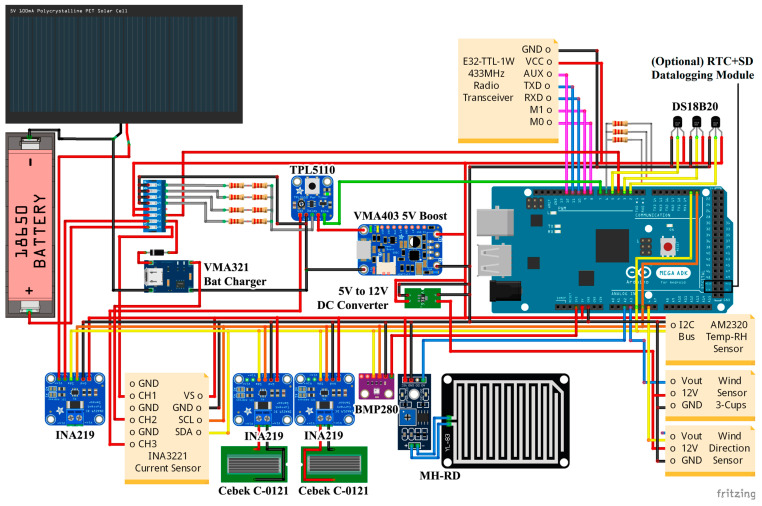
Transmitter electronics schematic featuring all available sensors and peripherals.

In its most complete version, the transmitter can include up to five temperature sensors (four air temperature sensors and one 9 cm black globe temperature sensor), a relative humidity sensor, a barometric pressure sensor, two solar irradiance sensors (one facing upwards and another facing downwards), a wind speed and wind direction sensor, a raindrops sensor, and various voltage and current sensors to ensure the correct operation of the device. A list of available sensors and characteristics is shown in Table 1, and the electronics schematic is shown in Figure 2.

Although ISO 7243 [25] requires globe temperature to be measured using a standardized 15 cm diameter globe, it allows for the use of alternative globe sizes. According to Vargas-Salgado et al. [51], it is possible to use a 9 cm diameter globe in a heat stress monitor, with an acceptable margin of error. This size is cheaper and easier to handle.

An Arduino microcontroller manages this system, being compatible with both Arduino MEGA and UNO boards. Thanks to the Arduino’s flexible and modular nature, the electronic circuit of the transmitter is prepared to connect additional sensors through the I2C and SPI buses. Therefore, it is compatible with a real-time clock (RTC) + SD card module that could provide offline data logging if necessary, avoiding the need for a receiver in some applications. Likewise, the transmitter also allows us to easily disconnect those sensors not needed in each case, helping to reduce manufacturing costs.

The transmitter has been designed to operate autonomously and wirelessly indoors and outdoors. It is powered off-grid by up to four 3.7 V, 2600 mAh, 18650 Lithium-ion batteries. It also includes a 3.5 W, 6 V, 165 × 135 mm photovoltaic (PV) panel that charges the batteries during the day. To save energy between measures, a TPL5110 timer switches off the device and puts it into standby mode. It can also be powered from the grid using a 5 V DC charger if needed.

#### 3.1.2. Transmitter Firmware

**Figure 3:**
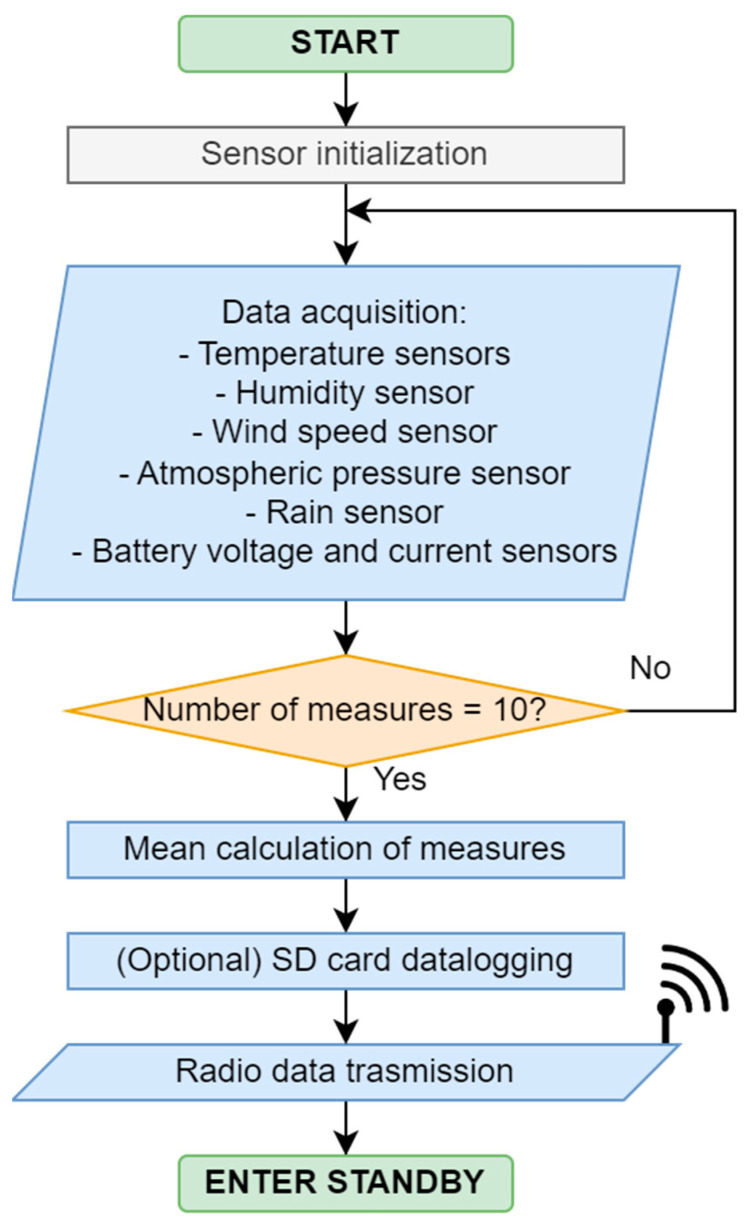
Transmitter firmware flow diagram.

The Arduino MEGA microcontroller has been programmed in C++ using the Arduino IDE 2.2.1, following the flow diagram described in Figure 3. The timer switches on the transmitter and runs the Arduino program for a certain amount of time which is 15 min by default. This interval can be selected using a switch between 1, 15, 30, and 60 min. The program takes 10 measures (one per second) from every sensor and calculates the arithmetic mean, filtering the error values. Then, it sends the final values and the device’s ID through an E32-TTL-1W 433 MHz radiofrequency module with an antenna, manufactured by EBYTE and acquired in the local market in Valencia, Spain, with a maximum nominal range of 7500 m in line of sight. This system allows for unlimited transmitters to send data to one receiver within the radio range. Finally, the timer switches off the device to save energy and waits for the next cycle.

#### 3.1.3. Transmitter Box

The device has been assembled into a rainproof custom PLA plastic 3D-printed box. This box has been designed to accommodate all the external sensors: the anemometer, the black globe, two irradiation sensors and the raindrops sensor, as well as the radio antenna and the solar panel in an optimal tilt angle of 48°. A methacrylate semi-sphere protects the upper irradiation sensor to avoid sensor degradation and minimize dirt accumulation. It also includes a small solar radiation shield for the temperature and humidity sensors. The DS18B20 sensors allow for two configurations to measure air temperature embedded into the device and measure air or surface temperature through an up to 100 m cable.

**Figure 4:**
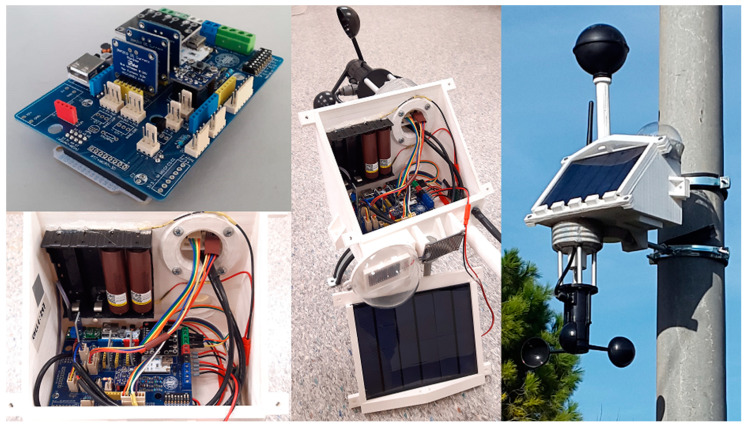
Overview of the transmitter box in various manufacturing and installation stages.

The box was coated with exterior acrylic paint and a clear varnish to endure extreme weather conditions and prevent PLA degradation. The joints were also sealed with silicone, and the radiation shield was covered with an insect screen. The transmitter can be installed with metal clamps and screws on walls or light poles in study locations. Figure 4 shows the different manufacturing and installation stages of a transmitter box.

### 3.2. Receiver and Database

**Figure 5:**
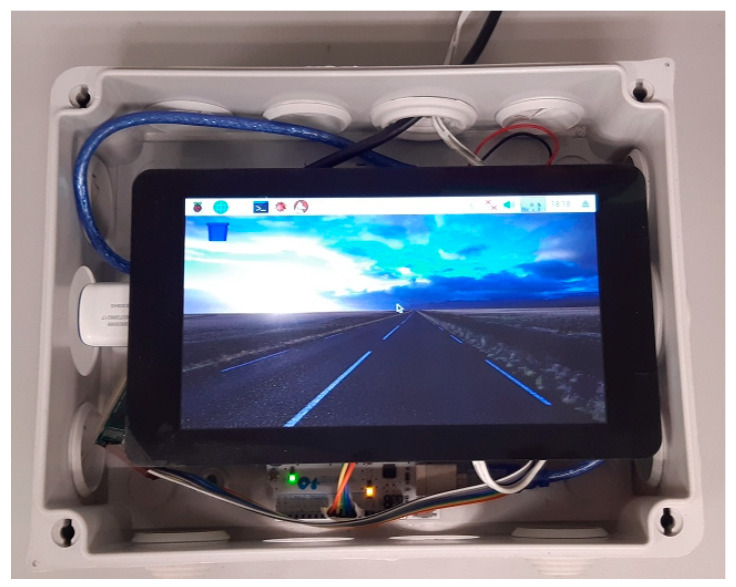
Receiver installed and running.

**Figure 6:**
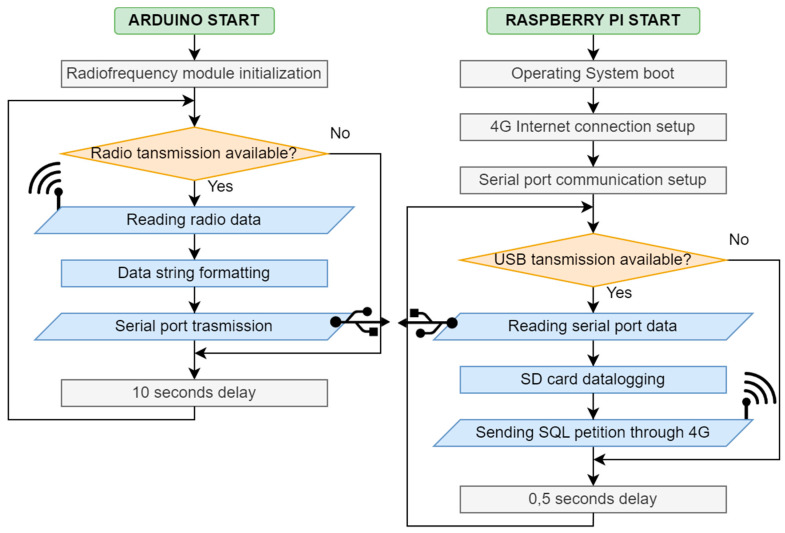
Receiver firmware flow diagram.

Several parts form the receiver (Figure 5). First, an Arduino UNO microcontroller connected to another 433 MHz radio frequency module receives the data sent by every transmitter. Then, following the flow diagram in Figure 6, it sends this data via serial communication through a USB cable to a Raspberry Pi 3 board. This board works on a Debian-based operating system, which runs several Python and Linux command scripts that manage the internet connection through a 4G USB Modem and upload the data to a SQL database hosted in the cloud. Data is also saved on a microSD card for a local backup. Each radio module is configured with a unique ID that identifies the receiver. This way, every transmitter sends data to just one receiver, allowing for unlimited transmitters to send data to up to 256 receivers in the same area thus avoiding duplicate measures.

The receiver is assembled into an electrical connection box with a 7″ LCD touchscreen to navigate the OS. It needs to be installed where it can be powered from the grid with a USB power supply. The receiving radio antenna can be connected to the box through an extension cable and installed outdoors to improve range.

All the data collected by the transmitters’ sensors is stored in an online SQL database. Users can access the database to display and download the desired data. Table 2 shows all the variables available. The transmitter and the database filter all the error values and measures. Failed radio transmissions are logged into a separate table, and incorrect or missing measures are displayed with specific error values (by default, ‘32,767’) that can be easily filtered afterwards.

## 4. Results and Discussion

### 4.1. Application Case: Benicalap (Valencia)

**Figure 7:**
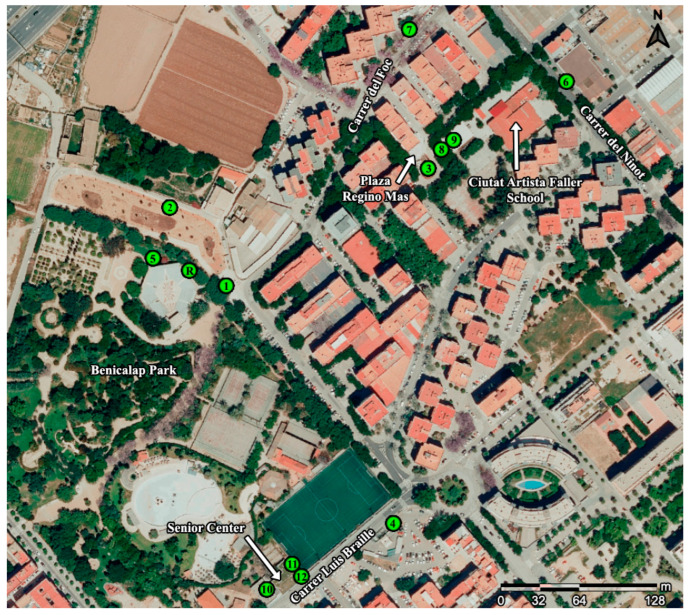
Location of HSM devices installed in Benicalap-Ciutat Fallera district. Source: Own creation based on an image obtained from Google Maps in June 2021.

The system was tested in Valencia (Spain). Twelve transmitters and a receiver were installed in various locations within the Benicalap-Ciutat Fallera district. They were left to operate for more than one year, and the data was collected for analysis. The devices’ locations are shown in Table 3 and Figure 7.

As noted, in some cases, several devices are positioned in the same locations. The reason is to collect data from different environmental conditions within the same site, each serving a different purpose. For instance, in the senior center, HSM11 is located indoors. Meanwhile, HSM10 and HSM12 are installed outdoors on different roof sections. HSM12 will measure the effects of a planned green roof project, while HSM10 serves as a control. The same situation applies at the school with HSM8 outdoors and HSM9 indoors and with both devices installed on both sides of the same wall. The rest of the units are installed in various points of interest within the neighborhood, with different insolation levels or urban layout.

This application case is part of the Grow Green project, a European Union initiative. Its main objective is to tackle heat stress in urban environments through the use of nature-based solutions. Thus, several different actions have been planned in the Benicalap-Ciutat Fallera district, such as green roofs and walls, or to expand the green areas throughout the district. In this scenario, the HSM prototype plays a key role in the scientific research around the experimental validation of the solutions proposed.

### 4.2. Testing Results

#### 4.2.1. Wireless Data Transmission Test

**Figure 8:**
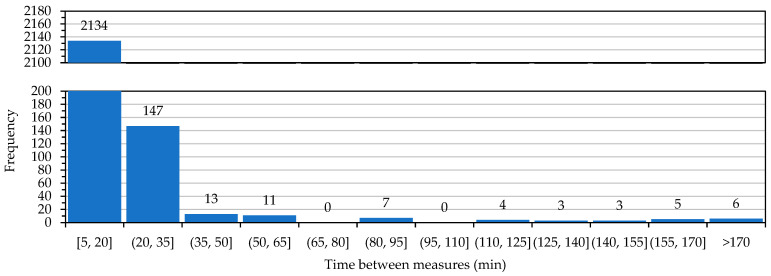
Histogram of elapsed time between successful measures for HSM1 in March of 2021.

Analyzing a month of data registered in the database, given that measures should be taken every 15 min, we can determine how many radio transmissions failed. Due to the slow dynamics of the studied parameters, such as temperature, it is not critical to lose some inputs. For that, we calculated in which time intervals the measures were taken to determine when a significant amount of data was lost. As an example, a histogram of elapsed time between successful measures for HSM1 is shown in Figure 8.

Comparing these results with devices in other locations, Table 4 shows data from three devices and two additional test units installed in Universitat Politècnica de València (HSMUPV) that cover short, medium, and long-range measures, in both urban and line-of-sight settings. Data shows at least 97.5% of the radio transmissions were successful at most every 35 min (that is, every 15 or 30 min). We can therefore approve the radio system and consider it valid. Furthermore, if this data is contrasted with the distance of every device to the receiver, it can be determined that there is no significant change in the efficiency. However, there is a mean reduction of 31% in transmission errors when the transmitter is in line of sight. With that, we can establish a maximum valid tested range for the radio system of 345 m for urban environments and 700 m in line of sight.

#### 4.2.2. Power Supply and Autonomy Test

The INA219 sensor measures the power consumption of the transmitter through its input current and voltage when the transmitter is switched on (about 20 s). The rest of the time, the TPL5110 timer switches off the transmitter to save energy. Two tests were carried out to obtain the transmitter power consumption and autonomy.

In the first test, a transmitter was left to operate in a laboratory, taking measures every minute until the battery was drained. As a result, it was obtained that the average power consumption of the transmitter while sending data was 0.641 W (33.33% of the time during the test) and 0.31 W in standby mode (66.66% of the time). The energy consumed to discharge the battery during the test (21.67 h) was 9 Wh. Thus, we obtain an estimated prototype battery life of around nine days per battery.

**Figure 9:**
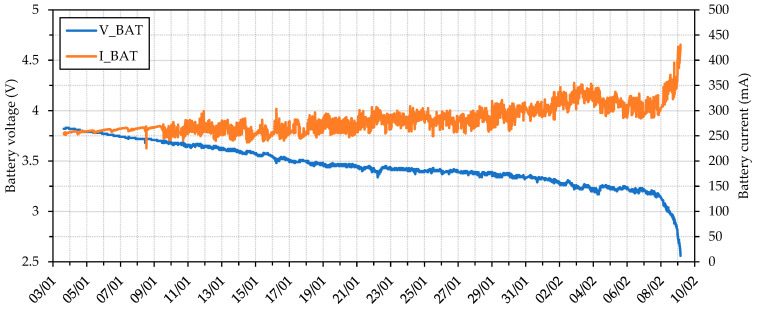
Battery discharge profile with four batteries measuring every 15 min without a PV panel. Data collected from HSM1 in real operating conditions from 3 January to 10 February 2019.

A second test was carried out to verify the device’s performance and energy consumption. It used data from a device installed in its final location under actual operating conditions, taking measures every 15 min with its PV panel disconnected. The test results revealed that using all the sensors, the device could operate autonomously for up to 36 days using four batteries without recharging (Figure 9).

**Figure 10:**
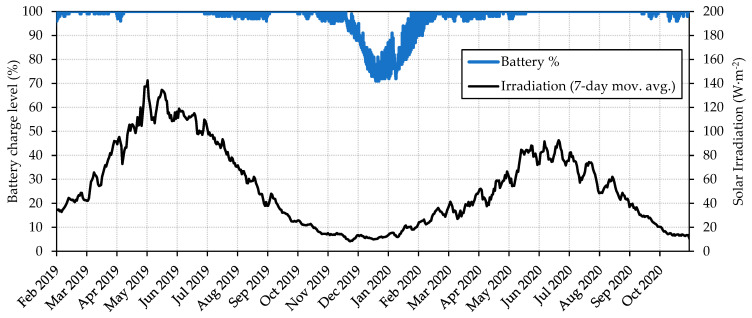
Battery state of charge and solar irradiation (7-day moving average). Data collected from HSM1 with PV panel in real operating conditions from February 2019 to November 2020.

This supports the results in Figure 10 which shows that, when the PV panel is connected, the battery state of charge is always above 95%, as long as the daily average solar irradiation is over 10 W·m^−2^. Even under long periods of solar irradiation under 10 W·m^−2^, it is able to maintain the charge level above 70%. This ensures an unlimited battery life for the transmitter, working with no interruptions or energy shortages even on cloudy days, in low light regions, under dense foliage, or in the event of a faulty PV system.

#### 4.2.3. Device Robustness

Several transmitters have been thoroughly tested in an urban environment for over a year or more. They have endured harsh weather conditions, including temperatures from −5 °C to 45 °C, strong winds, heavy rain, and long periods of intense solar irradiation.

Previous prototypes of transmitters had several flaws that required continuous updates. The most critical problems were water-related issues. Although 3D-printed PLA parts are easy to print, they are not water-tight because of the nature of fused deposition modelling. Also, PLA is not UV resistant; the material degrades after prolonged sunlight exposure [52]. Some boxes even broke when trying to open them. Thus, water filtrations during light and heavy rain were common, which led to corrosion and electrical problems. Another frequent problem was insects entering the box through the sensor radiation shield and creating dirt in the electronic circuit.

The proposed modifications included redesigning the box to minimize potential water infiltration paths. All 3D-printed components were coated with exterior acrylic paint and transparent varnish to protect them against direct sunlight exposure and enhance the seal. Critical joints were sealed using PTFE tape and silicone. An insect screen was installed within the radiation shield to deter insect entry. Subsequently, rigorous laboratory testing involving a continuous flow of water falling on the box for five minutes confirmed the absence of water infiltration issues, with no further reported problems in this regard.

It is important to note the transmitter’s limitations concerning sensor robustness. Sensors were selected for accuracy, prioritizing cost-effectiveness. However, certain sensors, such as the DHT22, AM2320, and BME280 relative humidity sensors, exhibited problems after prolonged exposure to high humidity. Fortunately, these sensors are affordable and readily replaceable. Another concern is the solar irradiation sensor (Cebek C-0121), which experiences degradation over time, manifesting as yellowing and dirt accumulating on its surface, affecting measurement accuracy. To mitigate this issue, the proposed solution involves enclosing the sensor within a methacrylate dome.

No breakdowns were reported in the most recent transmitter iterations due to weather conditions (after one year of testing). However, regular sensor checks during annual maintenance and timely necessary replacements are advisable.

### 4.3. Sensor Data Collected

**Figure 11:**
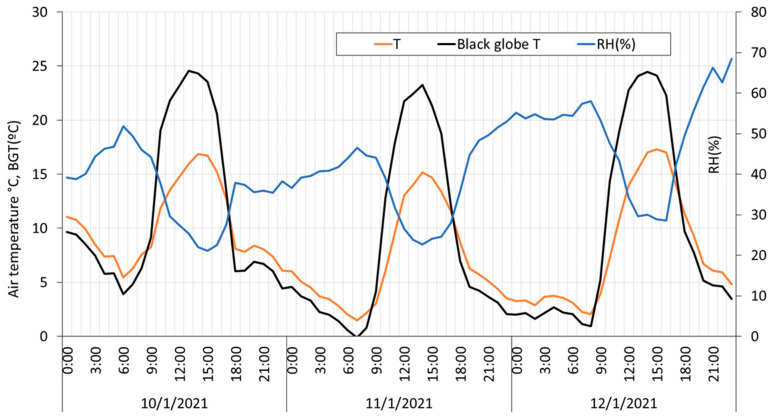
Air temperature, black globe temperature, and relative humidity data. Collected from HSM1 from 10 January to 12 January of 2021.

**Figure 12:**
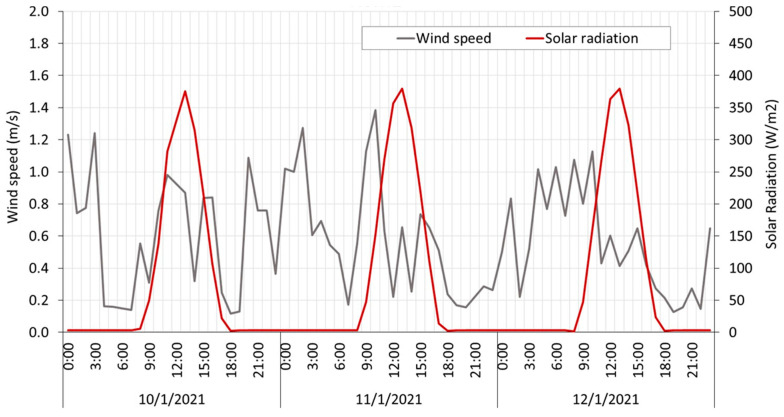
Wind speed and solar radiation data. Collected from HSM1 from 10 January to 12 January of 2021.

Data extracted directly from the database allows us to plot the main parameters under study. Figure 11 shows air temperature, black globe temperature, and relative humidity data, and Figure 12 shows wind speed and solar radiation data collected from HSM1 from 10 January to 12 January of 2021.

**Figure 13:**
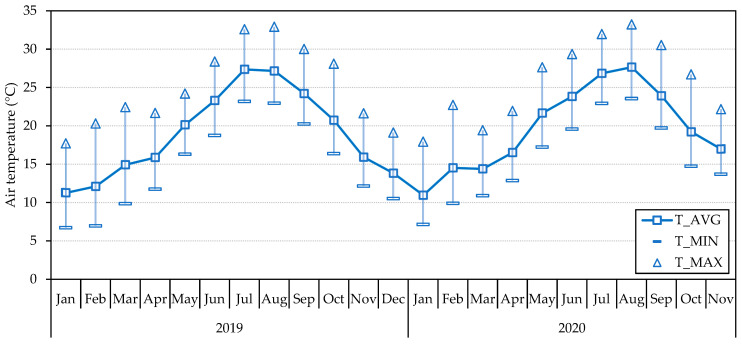
Monthly average of minimum, average, and maximum daily temperatures from HSM3 in 2019 and 2020.

We can also plot the annual temperature variations by gathering data from various months. Figure 13 shows the monthly maximum, minimum, and average air temperature calculated from the daily averages from HSM3 from January 2020 to November 2021.

The system successfully operated for nearly two years, diligently capturing regular daily and seasonal variations in air temperature patterns, humidity levels, black globe temperature, and solar radiation. To validate its accuracy, temperature and humidity values were meticulously compared with the data from the nearest reference weather station (Viveros, AEMet ref. 8416, N 39°28′50″ W 0°21′59″). The outcomes revealed a mean absolute error of approximately 1.15 °C for temperature and 8.01% for humidity. However, it is fundamental to acknowledge that the reference station, situated around 3 km from the HSM sensor, operates under distinct conditions. Furthermore, the reference station provides hourly data, while the HSM delivers information at a higher temporal resolution every 5 min.

Consequently, the comparison’s relevance is somewhat limited due to the temporal mismatch and differing environmental contexts. Despite these considerations, a cross-verification of temperature and humidity sensors was conducted. The BME280 and AM2320 relative humidity sensors, along with the DS18B20 and AM2320 temperature sensors integrated into the same device, were scrutinized (refer to Table 1). The analysis yielded an average absolute error of 0.25 °C and 5.96% for temperature and humidity, respectively, well within the nominal accuracy range. This underscores the HSM’s capabilities in ensuring measurement reliability, particularly through sensor redundancy. The redundancy mechanism significantly enhances the system’s robustness, contributing to its overall effectiveness in providing accurate and dependable environmental data.

### 4.4. Heat Stress Indices Calculation

**Figure 14:**
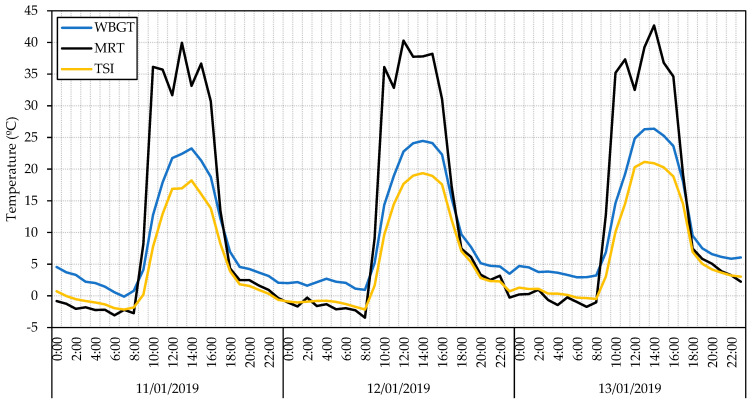
WBGT, MRT, and TSI heat stress index calculation from three winter days. Data collected from HSM1 from 10 January to 12 January 2019.

Using the data collected by the HSM system, we can estimate the heat stress indices described in the Methodology (see Section 2.1). Figure 14 shows the results of calculating the WBGT, MRT, and TSI index for HSM1 from 10 January to 12 January 2019.

### 4.5. Total Cost of the Measurement System

Table 5 describes a detailed list of the cost of materials for an HSM system. This includes the individual cost of every transmitter and receiver and its components plus an extra 10% added for tooling use and production losses. Manufacturing costs as well as time and resources invested in design, development, and testing are not included.

The total cost per device of the whole HSM system is reduced as more transmitters are included, as they can communicate with the same receiver. This is especially convenient for study locations like urban environments where many measuring points are needed in a reduced area. Finally, it is worth noting that the final cost of the system can be significantly reduced depending on the application. The list of sensors can be reduced to the most strictly necessary ones, the power needs may be covered with only two batteries, and the 4G modem and data plan may not be needed at all if the receiver is installed in a site with Wi-Fi connection.

As Table 6 shows, the HSM system’s final cost per node is significantly lower than other commercial solutions [53,54], and it has some additional useful features that mean an advantage over its competitors.

Finally, if we calculate the total cost of the HSM system described in the application case (see Section 4.1), with a 12-node network of transmitters and one receiver, we obtain a total of EUR 2429.54. This allows us to cover large and dense areas while maintaining a very competitive price.

## 5. Conclusions

This study aimed to design, build, commission, and test a low-cost Heat Stress Monitoring (HSM) system capable of measuring in an urban environment. The devices have been tested for more than one year. The HSM system consists of two parts: a series of transmitter devices that measure the environmental parameters through sensors and send the data through radio transmission, and a receiver device that receives the data from the transmitter and sends it to a cloud-based database.

The key environmental parameters the HSM system measures include air temperature, relative humidity, wind speed, atmospheric pressure, solar irradiation, and black globe temperature. The transmitter devices can be powered off-grid by a PV panel and Li-ion batteries or connected to the grid through an AC charger. They include a timer that puts the device in standby mode between measures to save energy. This gives an autonomy of up to 36 days per charge, although the PV panel can provide unlimited autonomy.

The HSM system successfully provided the parameters required to estimate heat stress indices such as WBGT, MRT, and TSI using the data collected by the devices. The HSM system can operate indoors and outdoors, and is designed to be replicable, open-source, and low-cost. The material cost for a 12-point network is around EUR 190 per measuring point, significantly lower than other commercial solutions.

The devices have demonstrated resilience in challenging weather conditions but are not without limitations. Previous prototypes were prone to water-related issues, with water infiltration during rain leading to corrosion and electrical problems. Insects entering through the sensor radiation shield caused dirt buildup in the electronic circuit. While design improvements have addressed these problems, the transmitters still have limitations related to sensor robustness. Some sensors, like relative humidity and solar irradiation, may degrade over time, particularly in high-humidity environments or due to yellowing and dirt accumulation. Replacing these sensors during periodic maintenance is recommended to maintain accuracy and functionality.

Further developments of the HSM system will focus on improving long-term durability by adopting more robust sensors, scaling up manufacturing to bring down costs even further, and exploring the possibility of giving 4G capabilities to the transmitters, eliminating the need for a receiver and making the system even more versatile.

The HSM system developed in this study is an innovative and cost-effective solution for monitoring heat stress in urban environments. The system’s ability to measure key environmental parameters, estimate heat stress indices, and operate in extreme weather conditions makes it an attractive option for researchers, city planners, and public health officials concerned with mitigating the effects of heat stress.

## Figures and Tables

**Table 1:** List of available sensors in the transmitter and its characteristics.

Component	Qty	Variable	Range	Output	Resolution	Accuracy	Price
DS18B20	3	Temperature	−55~125 °C	Digital	12 bits	±0.5 °C	EUR 0.90
AM2320	1	Temperature	−40~80 °C	Digital	0.1 °C	±0.5 °C	EUR 1.65
		Relative Humidity	0~100%	Digital	0.1%	±3%	
BME280	1	Air pressure	300~1100 hPa	I2C Bus	0.16 Pa	±1 hPa	EUR 0.65
		Relative Humidity	0~100%	I2C Bus	0.01%	±3%	
		Temperature	−40~85 °C	I2C Bus	0.1 °C	±1 °C	
JL-FS2	1	Wind speed	0 (0.4–0.8)~30 m/s	0~5 V	0.1 m/s	±3%	EUR 32.30
HYXC-FXV	1	Wind direction	0~360° (from 0.5 m/s)	0~5 V	22.5°	±3%	EUR 32.05
INA219	3	Voltage	0~26 V	I2C Bus	12 bits	±1%	EUR 0.80
		Current	±3.2 A	I2C Bus	0.8 mA	±1%	
INA3221	1	Voltage	0~26 V	I2C Bus	12 bits	±1%	EUR 1.95
		Current	±3.2 A (3 Channels)	I2C Bus	0.8 mA	±1%	
MH-RD	1	Raindrops	0.1~2 MΩ	0~4.2 V	10 bits	N/D	EUR 0.40
Cebek C-0121	2	Solar irradiance	0~1000 W/m^2^	0~300 mA	0.8 mA	±1%	EUR 32.62

**Table 2:** List of variables accessible from the database.

Variable ID	Unit	Type	Description
Receiver	N/A	int	Receiver ID number (transmitters only send data to a specific receiver ID).
TX_ID	N/A	int	Transmitter ID number.
BAT_LV	%	float	Battery charge level calculated from V_BUS_V.
HR_BME	%	float	Relative humidity from BME280 sensor.
TEX_BME_C	°C	float	Air temperature from BME280 sensor.
WBGT_DS_C	°C	float	Black globe temperature from DS18B20 sensor.
TX_DS_C	°C	float	Air temperature from DS18B20 sensor.
TBOX_DS_C	°C	float	Additional DS18B20 sensor, usually measures transmitter box temperature.
HR_AM	%	float	Relative humidity from AM2320 sensor.
TEX_AM_C	°C	float	Air temperature from AM2320 sensor.
V_WIND_CUP	m/s	float	Wind speed from JL-FS2 sensor.
DIR_WIND	N/A	string	Wind direction from HYXC-FXV. Up to 16 values (N, NW, SSE, etc.).
PATM_Pa	Pa	float	Atmospheric pressure from BME280 sensor.
IRR_UP_Wm2	W/m^2^	float	Solar irradiation from Cebek C-0121 sensor facing upwards.
IRR_DOWN_Wm2	W/m^2^	float	Reflected solar irradiation from Cebek C-0121 sensor facing downwards.
RAIN	%	float	Estimated amount of rain (from none to full-wet) from MH-RD sensor.
V_BUS_V	V	float	Power supply voltage (batteries and solar charger) from INA3221 sensor.
V_PV_V	V	float	PV panel voltage from INA219 sensor.
I_BAT_mA	mA	float	Battery current from INA219 (negative means batteries are charging).
I_CH_mA	mA	float	Solar charging current from INA219 sensor.
I_IN_mA	mA	float	Current consumed by the Arduino and electronics from INA219 sensor.
I_PV_mA	mA	float	PV panel current from INA219 sensor.

**Table 3:** List of HSM devices installed in the Benicalap-Ciutat Fallera district.

ID	Location	Coordinates	Distance	Installation
Receiver	Benicalap Park: Office	39°29′55.2″ N 0°23′46.7″ W	-	25 October 2018
HSM1	Benicalap Park: Entrance	39°29′54.8″ N 0°23′45.6″ W	33 m	9 January 2019
HSM2	Benicalap Park: Copse	39°29′57.0″ N 0°23′47.5″ W	70 m	9 July 2021
HSM3	Plaza Regino Mas	39°29′57.6″ N 0°23′38.3″ W	214 m	9 January 2019
HSM4	Calle Luis Braille	39°29′48.4″ N 0°23′40.2″ W	260 m	9 January 2019
HSM5	Benicalap Park: Theatre	39°29′55.5″ N 0°23′48.0″ W	34 m	11 December 2018
HSM6	Carrer del Ninot	39°30′00.0″ N 0°23′33.6″ W	345 m	9 January 2019
HSM7	Carrer del Foc	39°30′01.5″ N 0°23′38.7″ W	271 m	17 April 2019
HSM8	School: Outer wall	39°29′58.4″ N 0°23′37.8″ W	235 m	20 May 2019
HSM9	School: Inner wall	39°29′58.4″ N 0°23′37.8″ W	235 m	1 June 2019
HSM10	Senior Center: Roof	39°29′46.7″ N 0°23′43.9″ W	263 m	10 May 2019
HSM11	Senior Center: Indoor	39°29′47.1″ N 0°23′43.2″ W	258 m	10 May 2019
HSM12	Senior Center: Roof	39°29′47.1″ N 0°23′43.2″ W	258 m	1 June 2019

**Table 4:** Relative frequency of elapsed time between successful measures and final radio transmission efficiency, depending on the distance to receiver.

Time Between Measures (min)	Relative Frequency (%)				
	HSM1	HSM3	HSM6	HSMUPV6	HSMUPV7
<20	91.47%	91.72%	91.37%	92.31%	91.32%
<35	97.77%	97.92%	97.53%	99.24%	98.19%
<50	98.33%	98.51%	98.30%	99.58%	98.74%
<65	98.80%	98.78%	98.83%	99.70%	99.34%
<80	98.80%	99.10%	99.31%	99.83%	99.73%
<110	99.10%	99.37%	99.55%	99.96%	99.95%
<140	99.40%	99.69%	99.76%	99.96%	100.00%
<170	99.74%	99.69%	99.80%	99.96%	100.00%
Distance (m)	33	214	345	570 (*)	700 (*)
Efficiency (%)	84.21%	84.31%	84.11%	89.20%	89.17%

(*) In line of sight.

**Table 5:** Detailed budget for the transmitter and receiver materials.

Item	Cost
Transmitter: Sensors	EUR 81.03
Transmitter: Electronic components	EUR 58.12
Transmitter: Lithium batteries (4 units)	EUR 17.55
Transmitter: Mechanical parts	EUR 17.19
Transmitter: 3D printed parts	EUR 12.87
Transmitter Total	EUR 186.76
Receiver: Electronic components and other parts	EUR 144.95
Receiver: SIM Card data plan (24 months)	EUR 26.40
Receiver: 4G USB Modem	EUR 17.07
Receiver Total	EUR 188.42

**Table 6:** Comparison chart between the HSM system and other commercial solutions in terms of cost per node and other key features.

	HSM	HD32.3A-CV	Davis Vantage Pro2™ Plus
Dedicated heat stress measuring.	✕	✓	✕
Black globe temperature sensor	✓	✓	✓
Solar irradiation sensor	✓	✕	✓
Customizable and modular	✓	✕	✓
Data storage	Local + Online database	Local	Wireless display (300 m)
Off-grid power supply	Battery + PV panel	✕	Battery + PV Panel
Cost per node	EUR 190	EUR 3473	EUR 2325

## Data Availability

Part of the data are contained within the article, the complete set of data are not publicly available.
